# Satisfaction With Web-Based Healthcare Content in Cancer Survivors: A Cross-Sectional Survey

**DOI:** 10.3389/fdgth.2020.578792

**Published:** 2020-12-16

**Authors:** Akiko Hanai, Tappei Morino, Yuki Shinohara, Tomoki Aoyama, Tadao Tsuboyama

**Affiliations:** ^1^Human Health Science, Graduate School of Medicine, Kyoto University, Kyoto, Japan; ^2^Medical Sciences Innovation Hub Program, Institute of Physical and Chemical Research (RIKEN), Yokohama, Japan; ^3^School of Health Sciences, Bukkyo University, Kyoto, Japan

**Keywords:** cancer survivors, e-health, m-health, web-survey, self-management

## Abstract

Health-related web content is constantly increasing, and cancer survivors use it to manage their health and activities of daily living. However, the actual usage of and satisfaction with web contents among cancer survivors is unclear. Therefore, we conducted a web-based cross-sectional survey to understand the satisfaction with web content in those cancer survivors who use the Internet to cope with their anxiety/stress, sleeplessness, or cognitive difficulties. The survey questionnaire was e-mailed to 1.2 million voluntary registrants at a research company. Cancer survivors who accessed any content via the Internet and experienced anxiety/stress, sleeplessness, or cognitive difficulties were included in the study. Out of the 412 survivors who completed the survey, 357 experienced some degree of anxiety or stress, 258 experienced sleeplessness, and 161 experienced some cognitive difficulties, such as forgetfulness or lack of attention. They used web contents to record their health or (*n* = 205), relieve their anxiety or stress (*n* = 238), and devise activities of daily living (*n* = 232) during cancer therapy, including surgery, chemotherapy, and radiation. The web contents included “interactive contents” (users engage with the web content by responding to it in some form), “non-interactive contents” (information medium without any user engagement), “web-storage,” or “scrolling.” Multivariate logistic regression revealed greater satisfaction with “interactive contents” in cancer survivors. This reflects that the sharing of personal experiences as well as objective information should be considered to create satisfying and effective web contents.

## Introduction

Web-based medical content is rapidly increasing along with the patients' and caregivers' demand for web-based medical information. A recent systematic review suggests that approximately half of cancer patients trust the Internet as a source of cancer-related information, and explore websites for information about their illness (1). In Japan, cancer-related questions on websites dedicated to question and answer services are increasing, and half of the questions on these websites are about cancer treatment and diagnosis (2).

Cancer survivors use the Internet not only for information seeking, but also for self-management, including taking care of oneself without the help of any healthcare professionals. Useful web contents, such as online community sites, self-management healthcare applications, or cognitive behavior therapy tools are available (3–5). Additionally, the app with the highest number of active monthly users in Japan is a menstrual cycle management app (6), indicating that the need for self-management web contents and the frequency of their use will continue to increase. Systematic reviews suggest that combining the internet-based self-management programs with usual care improves the quality of life and alleviates psychological distress (7, 8). Internet usage plays a crucial role in treatment decision-making and self-management (9), and is expected to become an effective medical intervention tool for psychological stress management.

However, the emergence of communication technologies and greater access to health information does not ensure the quality of information-seeking experiences. A 2014 review of research evaluating the quality of online information resources revealed variability in the quality of information available, citing concerns about inaccuracy, incompleteness, and commercial bias (10). Furthermore, only about half of the population has the knowledge and skills necessary to access and differentiate between the massive amounts of web-based data (11). Mobile health (m-Health) has continuously been used as a method in several researches to improve health outcomes, and a review of 12 randomized control trials of m-Health interventions, including pre-programed reminders, concluded m-Health to contribute to statistically significant improvements in several health outcomes (12).

Internet services offering information support for cancer survivors are increasing, but the probability of accessing health-related information without frustration has not changed significantly over the last decade (13). While a few researchers have addressed the issue of Internet usage among cancer survivors, evidence regarding how cancer survivors use web contents for self-management is scarce. Accordingly, the objective of this study was to investigate the satisfaction related to web contents for self-management in cancer survivors.

## Methods

### Recruitment

A total of 1.2 million people registered on the Internet research monitor, Macromill Monitor (http://www.macromill.com/global/index.html), and agreed to participate in any health-related surveys on registration. Invitation emails to participate in the present study were sent to people who had registered as cancer survivors and had undergone cancer treatment. If the participants understood the details of the survey and clicked the URL link to the survey website, they were directed to a web-based informed consent page, after agreeing to which a screening survey started. The inclusion criteria were as follows: over 20 years of age; Internet user; experience of cancer treatment; and experience of using Internet sites or applications during cancer treatment either for management/recording of treatment and health status, relief from stress and anxiety, and/or devising activities of daily living during the treatment. This non-invasive research was approved by the ethics committee of Kyoto University Graduate School and Faculty of Medicine (R0464).

### Data Collection

The survey was conducted from March 9 to 11, 2016, in Japan. Participants who fulfilled the inclusion criteria were asked to complete a questionnaire with questions on the following:

#### Demographic Characteristics

Data related to age, gender, occupation, family, economic status, type of cancer, and treatment were collected.

#### Status of Anxiety or Stress, Sleeplessness, and Cognitive Difficulties During the Cancer Treatment Period

For assessing the presence and extent of symptoms related to stress, and disturbances in sleep and cognitive functioning, the participants had to choose from among the following options: “None,” “It did not affect my daily life,” “I had problems with my housework or professional work,” “I had difficulties in carrying out daily activities, such as eating or going to the toilet,” “I experienced severe dysfunction and consulted a hospital or specialized institution,” and “Others.”

#### Purpose of Use

This included “health records and diary of treatment,” “relieve anxiety or stress,” or “devising activities of daily living during the treatment” along with the names of the web contents used during the period of cancer treatment for each of these purposes. Multiple answers were allowed.

#### The Satisfaction With the Web Contents Used on the Participants' Physical and Mental Health

The participants were required to choose from the following options: “Very satisfied (great improvement in my work or daily living),” “Satisfied (a little improvement in my work or daily living),” “Partly Satisfied” (useful but no improvements in my work or daily living),” and “Not Satisfied” (I could not perceive any effect).”

#### Need for Web Contents During Cancer Treatment

This open-ended question required the participants to state, with reasons, what kind of web contents they wished for, regardless of whether these web contents were available for use.

### Data Analysis

In March 2015, the number of smartphone users in Japan was 50.06 million, and in January 2016, the most downloaded app in the health and fitness category was a life log app with 10 million downloads. Therefore, for ~20–60% of all users, life log apps would be a popular application category. When considering whether the subject was included in the top of the extracted categories, the tolerance was set within ±5%, the confidence level was set at 95%, and the population ratio was set at 50%. This yielded a required sample size of 385. The sample size was set to 400, assuming that ~3.5% of all the data would be inaccurate due to typographical errors. The data were reported using descriptive statistics and statistically analyzed. For evaluating satisfaction, we counted participant responses of both “Very satisfied” and “Satisfied” as “Satisfied.” Multivariate logistic regression analysis was conducted with the type of web content as independent variables and “Satisfied,” that is, satisfaction with the web contents as the outcome variable. Data analyses were conducted with R version 4.0.2. The statistical significance level was set at *p* < 0.05.

## Results

### Baseline Characteristics of the Participants

The demographic and epidemiological characteristics are shown in Table 1. Out of the 412 cancer survivors who completed the survey; 357 experienced some degree of anxiety or stress, 258 experienced sleeplessness, and 161 experienced some cognitive difficulties, such as forgetfulness or lack of attention (Table 2). The participants used web contents to record their health or therapy status (*n* = 205), relieve their anxiety or stress (*n* = 238), and devise activities of daily living (*n* = 232) during cancer therapy, including surgery, chemotherapy, and radiation.

**Table 1:** Demographics and epidemiological characteristics (N = 412).

	**Satisfied**	**Unsatisfied**	**Overall**
	**(*N* = 196)**	**(*N* = 216)**	**(*N* = 412)**
**Age (year)**			
Mean (SD)	56.2 (13.4)	58.4 (11.4)	57.4 (12.4)
Median [Min, Max]	56.0 [22.0, 84.0]	58.0 [30.0, 85.0]	57.0 [22.0, 85.0]
**Gender**			
Male	102 (52.0%)	103 (47.7%)	205 (49.8%)
Female	94 (48.0%)	113 (52.3%)	207 (50.2%)
**Married**			
Yes	153 (78.1%)	165 (76.4%)	318 (77.2%)
No	43 (21.9%)	51 (23.6%)	94 (22.8%)
**Personal Income**			
Under 20 thousand yen	70 (35.7%)	75 (34.7%)	145 (35.2%)
20–40 thousand yen	50 (25.5%)	63 (29.2%)	113 (27.4%)
40–60 thousand yen	28 (14.3%)	26 (12.0%)	54 (13.1%)
60–80 thousand yen	15 (7.7%)	16 (7.4%)	31 (7.5%)
Over 80 thousand yen	10 (5.1%)	12 (5.7%)	22 (10.8%)
Unknown	8 (4.1%)	10 (4.6%)	18 (4.4%)
Missing	15 (7.7%)	14 (6.5%)	29 (7.0%)
**Occupation**			
Public officials	5 (2.6%)	8 (3.7%)	13 (3.2%)
Managers and officials	4 (2.0%)	9 (4.2%)	13 (3.2%)
Office worker	21 (10.7%)	27 (12.5%)	48 (11.7%)
Engineer	12 (6.1%)	13 (6.0%)	25 (6.1%)
Other types of company employee	15 (7.7%)	15 (6.9%)	30 (7.3%)
Self employed	14 (7.1%)	15 (6.9%)	29 (7.0%)
Free lance	4 (2.0%)	4 (1.9%)	8 (1.9%)
Housewife (Househusband)	41 (20.9%)	44 (20.4%)	85 (20.6%)
Part-time job	19 (9.7%)	27 (12.5%)	46 (11.2%)
Student	1 (0.5%)	0 (0%)	1 (0.2%)
Otherwise	12 (6.1%)	9 (4.2%)	21 (5.1%)
Missing	48 (24.5%)	45 (20.8%)	93 (22.6%)
**Purpose of web contents use (Multiple answers allowed)**			
Record health or therapy status	104 (25.2%)	101 (24.5%)	205 (49.8%)
Relieve anxiety or stress	136 (33.0%)	102 (24.8%)	238 (57.8%)
Devising ADL	125 (30.3%)	107 (26.0%)	232 (56.3%)

*ADL, Activity of daily living*.

**Table 2:** Trouble during/after cancer treatment (number of patients).

	**Anxiety/Stress**	**Sleeplessness**	**Cognitive difficulties**
None	55	154	251
It did not affect my daily life	190	142	93
I had a problem with my housework or work	106	69	39
I had a difficulty in my daily living	85	42	28
I had a severe dysfunction and visit a hospital	39	32	21

The average age at the start of cancer treatment was 54.1 years [standard deviation (SD): 13.2, range: 19–85], while the current average age was 57.4 years (SD: 12.4, range: 22–85). Out of all the participants, 205 (49.8%) were women, 318 (77.2%) were married, and 290 (70.4%) were child-rearing. The cancer survivors experienced surgery (*n* = 389), chemotherapy (*n* = 176), and radiation therapy (*n* = 119).

### Satisfaction With Web Content

The reported web contents were categorized into four groups: “interactive contents” (users engage with the web content by responding to it in some form, such as social networking services (SNS), question and answer services, or blogs), “non-interactive contents” (information medium without any user engagement such as the official web sites of hospitals, committees, or governments), “web-storage” (applications for health record, diary, or note) or “scrolling” (simply surfing the unspecified web site).

To manage their anxiety or stress by searching for information related to their cancer treatment, 238 participants used web contents including symptom management or experiences of other cancer survivors. For health recording and keeping an updated diary of treatment, medical applications such as those on blood pressure management and medication notebooks were the most frequently used, followed by sites of pharmaceutical companies and the official cancer information website of the National Cancer Center. For lifestyle support, personal blogs and surfing the Internet were preferred; the use of applications providing the functions of recording and reminding, such as calendars and memos, was also reported for this purpose. Activities of daily living were devised by the participants by referring to advice from the question and answer services and medical applications for calorie calculation and food coordination. The satisfaction of the participants with each category of web contents has been reported using odds ratio (OR) and 95% confidence intervals (95% CI) in Table 3.

**Table 3:** Odds Ratio of satisfactions for web contents.

	**Record health or therapy status**		**Relieve stress or anxiety**		**Devising activities of daily living**	
	**Odds Ratio**	**95%CI**	**Odds Ratio**	**95%CI**	**Odds Ratio**	**95%CI**
Interactive web contents	1.31	0.57–3.00	3.95	2.21–7.09**	0.92	0.40–2.14
Non-interactive web contents	1.89	0.90–3.99	1.76	0.95–3.27	0.76	0.29–1.99
Web storage or diary apps	0.94	0.42–2.12	1.94	0.36–10.49	0.45	0.15–1.32
Strolling	1.03	0.45–2.37	2.02	0.30–13.55	0.64	0.26–1.58

** *P < 0.001 two-tailed ADL, Activity of daily living*.

We did not find any relationship between clinical/demographic factors and satisfaction with web contents among cancer survivors. None of them used healthcare applications whose effectiveness had been approved in a clinical trial.

The number of valid answers with regard to the need for web contents was 159, and the most frequently used keywords were “cancer” (*n* = 29), “blogs” (*n* = 23), “experience” (*n* = 13), “drug” (*n* = 12), “disease” (*n* = 5), “same disease” (*n* = 5), “hospital” (*n* = 5), and “treatment” (*n* = 5).

## Discussion

We analyzed the differences between patients who did or did not feel satisfied with their web experiences. Regardless of the background, including gender, age, or personal income, cancer survivors were significantly satisfied with interactive web contents such as SNS or private blogs, which they perceived as relieving their anxiety and stress. Such patients also tried to communicate with “comrades” who experienced similar situations and accompanied the survivors to peer support groups or real-world activities for cancer survivors. Conversely, the patients who were not satisfied with the web contents tended to use the Internet only to search for information about diseases, treatments, and hospitals. Although these differences may be affected by a potential response bias due to differences in the respondents' positive or negative attitudes, these results suggest that web contents that support interactive or continuous experiences correlate with the satisfaction of cancer survivors.

A review of m-Health clinical trials suggested that pre-set and tailored feedback on reported symptoms is a core component of effective m-health interventions (12). However, our data suggest that none of the participants used such evidence-based web applications, and that only a few cancer survivors used web-based monitoring or reminder systems for self-management, with which they were not well satisfied. As a review of electronic games for healthcare research showed, web contents create new styles of interventions include networking and communication, but how these styles compare with conventional face-to-face interactions is unknown (14). While we did categorize SNS, blogs, and question and answer sites as interactive content, further research will be needed to analyze the depth of interaction on such web-based interactive platforms because some survivors used them just as a source of information, without relying on them for the purpose of communication. To improve cancer survivors' quality of life, the needs matching strategy of m-health healthcare system should be researched for broad implementation.

This study had several limitations. The cross-sectional design used in this study reveals only correlations and does not provide any evidence regarding the effects of these web contents on the health status of cancer survivors. The web-based questionnaire also had several inclusion biases (motivation to use web contents, web literacy, and surveillance company-based recruitment), the response bias and recalling biases, while reporting biases were present in this original self-reported questionnaire which could not use for any diagnosis. Additionally, the missing data from the questionnaires appeared to be influenced by the Internet literacy of the respondents. Since it did not depend on age, it was difficult to adjust the effects of Internet literacy on the results. Furthermore, 43.7% of the participants in the present study were over 60 years of age. With the web questionnaire system, there were no missing data in the survey but there were several invalid answers (e.g., “forgot,” “could not understand,” or “uncertain meaning”), which might have been caused by poor Internet literacy. Considering that m-health interventions appear to be a viable health behavior change intervention modality for the youth (15), a survey of youth under 20 years of age may be expected to yield a different conclusion. The datedness of the data, which were collected over 4 years ago, was another limitation.

A scoping review suggested that future virtual survivorship care models may benefit from integrating existing health systems and services, repurposing common technologies, involving a variety of health professionals, and engaging patients and caregivers from diverse communities (16). While cancer patients prefer treatment cost information (17), the results of this study indicate that cancer patients who experienced distress or cognitive decline during the cancer treatment preferred searching not only for official information or systematically constructed web apps, but also for more interactive information, including the details of daily life and feelings during cancer treatment. This may be due to the fact that official sites mainly provide evidence-based information, whereas cancer survivors prefer information regarding personal lifestyles. As the current demand for Internet services in the medical fields increases, further research on web-based self-management to relieve anxiety or stress and manage sleepless or cognitive dysfunctions is needed.

## Data Availability Statement

The raw data supporting the conclusions of this article will be made available by the authors, without undue reservation.

## Ethics Statement

The studies involving human participants were reviewed and approved by Ethics committee of Kyoto University Graduate School and Faculty of Medicine. Written informed consent for participation was not required for this study in accordance with the national legislation and the institutional requirements.

## Author Contributions

AH and TT contributed to protocol writing, data collection, data analysis, and manuscript writing. TM, YS, and TA contributed to data analysis and manuscript writing. All authors contributed to the article and approved the submitted version.

## Conflict of Interest

The authors declare that the research was conducted in the absence of any commercial or financial relationships that could be construed as a potential conflict of interest.
